# Pregnant women’s experiences of social distancing behavioural guidelines during the Covid-19 pandemic ‘lockdown’ in the UK, a qualitative interview study

**DOI:** 10.1186/s12889-021-11202-z

**Published:** 2021-06-23

**Authors:** Emma Anderson, Amberly Brigden, Anna Davies, Emily Shepherd, Jenny Ingram

**Affiliations:** Centre for Academic Child Health, Bristol Medical School: Population Health Sciences, University of Bristol, 1-5 Whiteladies Road, Bristol, BS8 1NU UK

**Keywords:** Coronavirus, Pandemics, Covid-19, Pregnant women, Social distance, Infection control, Qualitative research, Behavioral research, Health-related behavior, Maternal health services

## Abstract

**Background:**

Covid-19 triggered the rapid roll-out of mass social distancing behavioural measures for infection control. Pregnant women were categorised as ‘at risk’ requiring extra vigilance with behavioural guidelines. Their understanding and ability to adhere to recommendations was unknown.

**Objectives:**

To complete a behavioural analysis of the determinants of recommended social distancing behaviour in pregnant women, according to the ‘capability, opportunity, motivation and behaviour’ (‘COM-B’) model to inform the development of recommendations/materials to support pregnant women in understanding and adhering to behavioural guidelines.

**Design:**

Qualitative interview study with pregnant women in the Bristol area (UK).

**Methods:**

Semi-structured telephone/videoconference interviews were conducted following a topic guide informed by the COM-B model, transcribed verbatim and subjected to framework analysis. Infographic materials were iteratively produced with stakeholder consultation, to support pregnant women.

**Results:**

Thirty-one women participated (selected for demographic range). Women reported adhering to social distancing recommendations and intended to continue. COM-B analysis identified gaps in understanding around risk, vulnerability, and the extent of required social distancing, as well as facilitators of social distancing behaviour (e.g. social support, motivation to stay safe, home environment/resources). Additional themes around detrimental mental health effects and changes to maternity healthcare from the social distancing measures were identified. Infographic resources (plus midwife report) addressing women’s key concerns were produced and disseminated.

**Conclusions:**

The COM-B model provided useful details of determinants of pregnant women’s adherence to social distancing behaviours. The confusion of what being ‘at risk’ meant and varying interpretation of what was expected indicates a need for greater clarity around categories and guidance. The loss of maternity care and negative mental health effects of social distancing suggest a growing area of unmet health needs to be addressed in future.

**Supplementary Information:**

The online version contains supplementary material available at 10.1186/s12889-021-11202-z.

## Background

### Covid-19 and social distancing

The coronavirus disease 2019 (Covid-19) outbreak was declared a public health emergency by the World Health Organization in January 2020 [1], and a global pandemic in March 2020 [2], due to the highly infectious nature of the disease and related risk of mortality. In the absence of pharmaceutical interventions, key strategies to prevent/limit the spread were mass behavioural measures for infection control [3]. These measures focused on social distancing behaviours as well as screening/isolation of positive cases and increased hygiene (e.g. handwashing).

The United Kingdom (UK) government provided such advice to its citizens, and in February gave strong social distancing behavioural advice for everyone to limit travel and contact with others and work from home if possible, though on a voluntary basis. The rapidly changing pandemic context led to the UK government implementing a nationwide social distancing ‘lockdown’ strategy on 23 March 2020 with the message “Stay Home, Protect the NHS [National Health Service], Save lives” [4]. The guidance stated that all members of the public should stay at home. Leaving the house was permitted for only four main reasons: shopping for necessities like food and medicine (limited frequency encouraged), to take exercise once per day only, for medical reasons (though people were asked to use telephone/online services where possible) or for essential work or where working from home was not possible (though many workplaces were closed). Social gatherings were not permitted, and when outside, people were to keep at least two metres apart from anyone not in their household. The guidance became law, with police given powers to ensure people followed the rules (e.g. to disperse groups and issue fines for noncompliance).

### Risk categories and pregnancy

In mid-March, the government classified pregnant women, along with people aged over 70 years and those with certain health conditions as ‘clinically vulnerable’ or ‘at risk’ of being more seriously affected by Covid-19, advising them to be particularly strict with following the social distancing behavioural guidelines. The government also classified those with specific medical issues into a further category of ‘extremely clinically vulnerable’ or ‘very high risk’, which included recipients of organ transplants, certain cancers, respiratory illnesses, and pregnant women with heart problems. This latter group were contacted by their General Practitioner (GP) from 23 March 2020 advising them to shield (not leave the house for any reason) for 12 weeks and they gained priority in online supermarket shopping delivery arrangements and volunteer support arrangements.

The social distancing guidance required unprecedented mass behaviour change, with advice shifting quickly as the pandemic situation progressed. The guidance specifically singled out pregnant women as an at-risk group in this context. To our knowledge, there is no existing research exploring pregnant women’s understanding of, and factors related to, the enaction of social distancing behaviours and no interventions designed to support these behaviours in this group.

### The COM-B model

The British Psychological Society’s Behavioural Science and Disease Prevention Taskforce advises using the Capability, Opportunity, Motivation- Behaviour (COM-B) model of behaviour change [5] to understand and facilitate the enactment of preventative behaviours in the context of the pandemic [6]. The COM-B model proposes that an individual must have sufficient capability, opportunity and motivation in order to enact a behaviour. Capability can be psychological (e.g. knowledge) or physical (e.g. skills); opportunity can be social (e.g. societal norms) or physical (e.g. environmental resources); motivation can be automatic (e.g. emotional and habitual) or reflective (e.g. beliefs and intentions). A COM-B analysis of determinants of behaviour can be used to diagnose deficient components to identify intervention targets to improve adherence to behavioural guidance.

COM-B has been successfully used in comparable research to explore the determinants of exercise uptake in new mothers to produce recommendations for how to improve physical activity [7], and in the context of the Covid-19 pandemic for exploring determinants of hand-washing behaviour in the general population to inform intervention development [8].

### Aims

This project aimed to explore pregnant women’s understanding of the behavioural restrictions and their perceived ability to comply, as well as the most concerning impacts of the measures. The overall aim was to develop and disseminate recommendations/materials to help pregnant women understand and adhere to the social distancing guidance.

## Methods

### Phase 1: COM-B analysis

#### Design

This was a qualitative interview study informed by the COM-B model [5], designed to explore pregnant women’s perceived capability, opportunity and motivation to follow the guidance on social distancing behaviours, and to identify barriers to, and facilitators of, these behaviours.

#### Recruitment and sampling

Women were invited opportunistically to express interest in taking part via The Bristol Post (local news media), university communications team, social media (Twitter and Facebook) and via local radio feature. In an attempt to increase ethnic diversity in the sample, Ujima Radio was targeted (a station for African-Caribbean and other BME communities) for study promotion, as well as community groups for Black, Asian and minority ethnic (BAME) groups on Twitter (e.g. Barton Hill/ Wellspring settlement, Ujima radio Twitter group). A statement was added to the study website to encourage women from BAME groups to participate in the study.

Participants were directed to the participant information sheet and an online expression of interest form, hosted on a study website [9]. Respondents were asked to provide their contact details and demographic characteristics for eligibility checking and sampling (see Additional file 1) and consent for a researcher to contact them to discuss the study. This form was captured on the Research Electronic Data capture (REDCap) system [10], a secure online data capture system designed exclusively for research studies. Information included the offer of £10 online shopping voucher for participating.

Eligible participants were pregnant women of any gestation, living in Bristol and the surrounding area, aged 18 or above (for full eligibility criteria see Table 1). Purposive sampling was applied to the pool of potentially eligible women who completed an expression of interest form, aiming for maximum diversity according to participant age, ethnicity, Index of Multiple Deprivation (IMD) by home postcode, gestation, and avoiding over-representation of those with medical/nursing training. Those selected were contacted by email, provided with a participant information sheet and invited to a research interview. A telephone/video call (depending on preference) was arranged for women wishing to participate, who were sent a link to an online consent form (captured on REDCap). At the start of each interview, the interviewer provided verbal information about the study, answered questions, and made final eligibility checks, ensuring informed consent was complete before proceeding to interview.

**Table 1:** Inclusion and exclusion criteria

Inclusion	Exclusion
Pregnant women (any stage of pregnancy)	No longer pregnant at time of interview
Access to telephone/ videocall facility and internet (for expression of interest form and receipt of online consent form)	No access to telephone/videocall facility/ internet
Home address in Bristol or immediate surrounding counties (North Somerset/West Wiltshire/South Gloucester)	Home address outside Bristol/immediate surrounding counties.
Aged at least 18 years	Aged less than 18 years
English-speaking	Unable to speak/understand English.*
Provision of informed consent	Women lacking capacity to provide informed consent (determined during phone/videocall prior to interview)

* While it would have been preferable to have an option to include non-English speakers, limited resources and rapid timeline prevented us from offering translation services

The aim was to recruit 30 pregnant women, aiming for data saturation of key COM-B model themes to answer the research question [11]. All methods were carried out in accordance with relevant guidelines and regulations.

#### Data collection

Qualitative interviews followed a topic guide structured around the COM-B model, which included a prompt to describe the social distancing guidelines presented on the Public Health England website at the time if these were unknown (See Additional file 2). Additional questions (outside of the COM-B model) asked participants: ‘What information would you find helpful and who would you like to hear it from?’ and; ‘Are there some other comments you would like to make on what we have talked about today?’. Interviews were conducted by all five authors, who have expertise in qualitative research, a background/interest in health psychology and interventions and an understanding of the COM-B model of behaviour change. Interviews were designed to last 30 min and were audio-recorded. Detailed interview notes were taken during or immediately after interviews using a structured template. On interview completion, participants were provided with emailed links to up-to-date social distancing guidelines [12, 13] and a £10 online shopping voucher. Audio-recordings were professionally transcribed verbatim, and transcripts were pseudonymised before full analysis.

#### Analysis

Framework analysis was applied to the data, following the seven steps outlined in Gale et al [14], which began with a rapid analysis of main themes (concurrent with data collection) prior to detailed analysis, as described in Table 2. Framework analysis was appropriate as the aim was specifically to identify subthemes within the COM-B model as a diagnostic framework to generate recommendations to help pregnant women follow government advice on social distancing behaviours. All transcripts were independently double-coded (by EA and AB) to ensure reliability, and discrepancies were resolved through discussion.

**Table 2:** Framework Analysis (seven step) method

Stage 1.	Transcription: Audio recordings were transcribed verbatim and pseudonymised. Detailed notes were taken by each interviewer (all authors), structured around the COM-B framework and additional questions.
Stage 2.	Familiarisation with the interview: Two authors (EA and AB) familiarised themselves with each interview by reviewing the detailed interview notes, and full transcript when available.
Stage 3.	Coding: EA and AB developed a matrix in an excel file, with columns representing each component of the COM-B framework plus the extra questions, and rows representing each participant. The initial rapid coding process involved systematically reading (and re-reading) the interview notes (and full transcripts where available) for each participant, assigning data to the relevant COM-B and extra question headings and identifying key subthemes within each component. Notes were made on relevant data which did not fit into the COM-B framework as potential inductive themes.
Stage 4.	Developing a working analytical framework: EA and AB met on two occasions to discuss in detail the findings for each participant (row) and the themes identified (column) as enabled by the framework analysis matrix, to agree the key themes, and produced a report of the initial findings.
Stage 5.	Applying the analytical framework: All full transcripts were imported into NVivo and the nodes function was used to set up the analytical framework established in step 4. Each transcript was coded by systematically assigning data to a node in the analytical framework. Authors swapped transcripts for coding so that all interviews were double coded.
Stage 6.	Charting data: Drawing on the full analysis in NVivo, EA created a table of the key themes with illustrative quotes, and reviewed it with all authors.
Stage 7.	Interpreting the data**:** During regular team meetings (10 meetings over the analysis phase), and via circulation of written materials, impressions and interpretations of the data, coding and the analytical framework were discussed and agreed. Rather than being a final stage, this process was ongoing throughout the analysis process.

### Phase 2: developing outputs

A report from our initial rapid analysis (stage 4, Table 2) identified key themes that were important to address in output/materials that could be used to support pregnant women during the pandemic. The stages outlined below were carried out with the aim of establishing what materials were needed, to whom they should be directed, and the appropriate delivery format/mode. Design and refinement of materials followed an iterative process involving stakeholders (community midwives, pregnant women, graphic designer).

#### Stage 1: consultation with expert group

The team consulted the University of Bristol’s Health Psychology and Interventions Group (HPIG) [15], presenting the initial findings and discussing next steps for informational output.

#### Stage 2: establishing outputs and delivery format

A single online meeting was conducted between study team members and two community midwifery staff from one NHS trust. A community midwife from a second NHS trust was separately consulted via email. This pragmatic approach was taken due to difficulties in convening staff during a busy period involving unusual working practices. Midwives were presented with the brief report of interview findings and were asked for their views about how the findings could be usefully conveyed to support women and midwives, and to whom those materials should be targeted (staff vs. women). Midwives were offered suggestions of mode of delivery of information including written materials, materials for a website and/or a video.

#### Stage 2: iterative design process

Following identification of the most appropriate target and modes of information delivery, the planned materials were developed iteratively with the assistance of a graphic designer (Oakshed.co.uk). Feedback was gained electronically from two pregnant women who had participated in the interviews, and community midwifery staff. Materials were checked for utility, clarity, content, format, layout, and colours used. Further iterations were reviewed by the research team in collaboration with the graphic designer to optimise appearance and functionality of the materials.

## Results

### Phase 1: qualitative interviews: COM-B analysis and main themes

Ninety-five women expressed an interest in participation, of whom 83 were eligible and 31 were selectively sampled (aiming for demographic diversity) and interviewed between 24 April–4 May 2020. Of the 31 women in the sample, 20 were primiparous; the age range was 24–48 years (mean 33), at the time of interview gestation ranged from 10 to 39 weeks (mean 24). Every IMD level [1–10] was represented and the ethnicity of our sample was: 24 (77%) White British, 7 (23%) other ethnicity as follows: 1 White European, 2 Asian, 1 Black, 3 mixed. Two had medical/nursing training.

The rapid analysis and team discussions during data collection indicated that reasonable data saturation for the COM-B framework themes was reached in our sample, as well as for the main additional themes, indicating that our sample was adequate and further recruitment was unnecessary.

An overview of the main themes identified are presented in Table 3. For the full analysis table with supporting quotations, see Additional file 3*.*

**Table 3:** Overview of thematic analysis according to the COM-B model

COM-B category	Themes identified
***BEHAVIOUR***	
Social distancing (in accordance with guidelines)	Adhering
	More extreme
	Slight deviations
***CAPABLITY – The individual’s physical and psychological capability to engage in the behaviour(s)***	
Psychological capability (understanding/ mental processes)	Knowledge and understanding of guidance around social distancing behaviours
	Confidence in ability to enact social distancing behaviours
Physical capability	Physical capability had little impact on social distancing behaviour
***OPPORTUNITY – Environmental factors influencing the behaviour(s)***	
Social opportunity	Social norms to comply with social distancing
	Household composition impacts on ability to enact social distancing
	Social distancing compromised by strangers in public spaces
Physical opportunity	Impacts of home environment and resources
	Work environment/ ability to work from home
	Shopping for essentials including preparation for the baby
	Healthcare appointments
***MOTIVATION – Individual internal factors that direct the behaviour(s)***	
Reflexive motivation	Motivated to adhere to social distancing guidelines
	Establishment of routines to enable social distancing
	Intentions to continue to adhere to guidelines
	Risks and balance of risks to determine behaviour
Automatic motivation	Emotional drivers of social distancing
	Automatic behaviours
***Beyond COM-B: cross-cutting themes***	
Isolation, mental health, and loss of maternity care	Isolation and mental health impacts
	Loss of maternity care – communication issues
	Loss of maternity care

#### Behaviour

Women reported adhering to the social distancing guidance to their best abilities, staying home as much as possible, limiting shopping trips, not allowing others in the house, going outside no more than once per day and staying at least two metres away from others when out. Many were taking extra precautions such as limiting their healthcare appointments and engaging in other behaviours that did not relate to social distancing but that aimed to reduce risk of exposure to Covid-19. These included washing and quarantining shopping, quarantining post or asking partners to do this before items came into the house. Six women in our sample were shielding (not leaving the house at all): two due to health issues in themselves or immediate household that increased risks from Covid-19; three were shielding believing this was the requirement when pregnant. Women reported few instances of breaching the social distancing rules, and breaches were minimal and carefully considered (e.g. driving a short distance to take exercise safely; isolation of home and parents’ households prior to moving in together for support).

#### Psychological capability

Women reported making efforts to access “credible” and “reputable” sources for information including government advice, BBC (British Broadcasting Corporation) news, Royal College of Obstetricians and Gynaecologists (RCOG)/ midwives, NHS, pregnancy apps/emails (e.g. Bounty), Tommy’s (online), select social media groups, contacts, scientific sources, and newspapers.

While mainly reporting good understanding of, and confidence in adhering to the guidelines, women reported a lack of clarity about why pregnant women were “at risk” and what it meant. Some felt it was precautionary, many wanted more explanation. Uncertainty around the risk category was evident in women’s different interpretations of what was expected of them: some interpreted it as requiring them to shield due to the ‘at risk’ status of being pregnant, while others stated that the guidance was no different for pregnant women from the rest of the population. Many felt the advice was confusing initially, though seemed clearer by the time of interview. Some details of the behavioural recommendations remained unclear, such as how to handle shared parenting between households, how to stay safe at work and whether to attend healthcare appointments for minor issues.

Psychological capability includes emotional ability to adhere to social distancing. While some women reported positive lockdown experiences (e.g. family time at home), many reported feeling isolated, low, and suffering loss of joy, which impacted on their perceived ability to sustain the behaviour. This was particularly acute when living alone and fully shielding.

#### Physical capability

Personal physical abilities had little relevance to adhering to social distancing recommendations, with just one example where pregnancy made it physically difficult to stop a toddler running close to others when outside.

#### Social opportunity

Women in our sample reported strong support from their immediate social circle to adhere to the social distancing guidelines, with friends, family and partners being strict about keeping them safe, particularly because of being pregnant. Women also described general social norms with ‘everyone’ adhering to the social distancing guidelines, or people generally expecting them to stay away (e.g. from work) due to being pregnant, with several gaining priority in their organisation’s homeworking/furlough schemes. By contrast, some women reported a minority of friends or family who did not adhere to the rules, though this did not influence their own social distancing behaviours. Women chose to reaffirm and explain the rules or keep a distance, with one participant reporting losing friends over this.

The household composition had a significant impact on women’s ability to maintain social distance. Many reported having supportive partners taking care of practical tasks, or other household members upholding social distancing practices. Others’ household composition brought added risks/challenges, such as co-parenting teenagers across two households, having to take small children to nursery, coping with the risks of other household members going out to work, or living alone, which made social distancing challenging both emotionally and practically.

Women reported being mindful of staying at least two metres away from others when out of the house, though many commented on being unable to control others’ behaviour, giving examples of people coming too close, or wanting a visible warning sign of being ‘at risk’.

#### Physical opportunity

Multiple physical opportunity determinants of social distancing behaviour were reported. Home environment and access to resources had a key impact: Participants recognised how lucky they were for resources they had (e.g. access to a garden or local green space, home exercise equipment, a car to avoid public transport), or for digital technology to facilitate social contact. Those in small flats or without gardens commented on how challenging it was to maintain social distancing and these women were hugely limiting their lives to adhere to the guidance and suffering negative mental health effects.

The ability to work from home enabled many women in our sample to maintain social distancing. Most felt these arrangements had been prioritised for them due to being pregnant. By contrast, one NHS employee reported having to push for work environment changes and reduced patient contact, while some chose to continue going to work if they could not work from home and judged the risk to be relatively low. Several women worried about future relaxing of the guidance (lockdown ending) and being expected to return to the workplace. Some who were unable to work from home chose to leave work, taking sick leave or early maternity leave to enable them to stay at home. These women reported facing financial consequences rather than put themselves at risk, though mortgage holidays, furlough schemes and workplace financial support made these decisions easier.

Shopping for essentials was challenging for all. Many commented on being unable to gain online shopping slots. Many relied on their partner or family members for regular food shopping or reported having to go themselves and feeling unsafe. One participant who expressed less anxiety than most, reported the weekly shop was the highlight of her week, while another who was single and shielding reported growing her own vegetables due to difficulties obtaining fresh food. Several women mentioned the difficulty with preparing for the coming baby – being unable to buy baby items from shops, online shops being out of stock, and having to consider how to manage handover of baby items from family members in a socially-distanced way.

Healthcare appointments were a key concern when it came to social distancing. Women worried about whether their ability to maintain distance when attending clinics and some chose to limit visits. Labour itself was concerning, with women knowing that they would be unable to maintain social distancing and worrying about it, with single women needing someone to take them to hospital. Some reported that antenatal appointments were the only times they had contact with other people and felt their social distancing abilities were compromised by needing to attend appointments. Participants with other children had to consider childcare arrangements, with one reporting asking a relative to shield for two weeks prior to her maternity appointment to facilitate childcare. Most participants acknowledged that changes to maternity care had enabled social distancing such as increased telephone consultations, limited face-to-face time, staff wearing personal protective equipment, spacing out waiting areas or enabling women to wait in their car to be called in to their appointment. Women reported that partners were not permitted to attend appointments as a social distancing measure and antenatal classes had been cancelled for the same reason. Some women sought alternatives online.

#### Reflexive motivation

Despite some of the challenges identified above, there was strong motivation to adhere to social distancing guidelines in our sample, with women adhering closely to the behavioural restrictions and many taking extra precautions. Women cited the safety of themselves and their baby as motivating factors as well as social responsibility, protecting the NHS and stopping the spread of the virus. Some mentioned a lower immune system during pregnancy making them more susceptible to infections; many expressed high motivation to avoid the virus near the birth. Some women mentioned their own higher risk status, such as a higher risk ethnicity or having comorbidities which made it even more important to adhere to the rules. No-one reported being motivated by the law or police sanctions. Many women reported having consciously established routines both to enable the maintenance of their social distancing lifestyle and to maintain sanity during lockdown.

When asked about intentions to continue, most women in our sample described their plans to continue with adhering to the behavioural advice, some wanting to continue with the current stringent measures if the lockdown was to ease, especially when nearing the time of birth. The birth event itself was pivotal, with some wanting to continue to maintain social distance afterwards to protect their new-born baby, and others expressing a need to ‘break the rules’ to gain support with the new-born baby or see people, weighing up the relative risks of this decision.

Decision-making and planning around social distancing behaviours often involved weighing up relative risks. Women described difficult decisional processes such as being unsure whether it was safer to attend the midwife appointment or avoid it, or whether exposure risk outweighed the mental/physical health effects of outdoor exercise. This was compounded by a lack of understanding around the reasons for being in the ‘at risk’ group. Planning arrangements around the birth were fraught as women grappled with how to plan for a parent to help look after existing children during labour or for support with the new-born, or if their partner became ill.

#### Automatic motivation

Fear was identified throughout interviews as a motivating factor for adhering to social distancing guidance. Fears focused on worries about catching Covid-19 near the birth date, or the partner becoming ill, as well as general risk to self and baby. Other emotional drivers of social distancing included guilt or an anticipated guilt of catching the virus due to going out, especially compounded by a sense that women felt capable of adhering to the rules. Conversely, the sadness and low mood some women experienced from their social isolation posed a challenge to adherence.

Unconscious processes and automatic behaviours are difficult to assess in a reflective interview, though some women reported that they were now automatically enacting social distancing and other protective behaviours (e.g. keeping away from strangers, handwashing and washing shopping).

#### Beyond COM-B: isolation, mental health and loss of maternity care

While some positive aspects of following the social distancing guidance were identified by women in our sample, such as spending more time with their immediate family or enjoying working from home, there were strong themes of social isolation and negative mental health across interviews. Pregnant women living alone (or only with small children) were particularly vulnerable to isolation and mental health effects. Pregnancy was seen as a time when women would normally seek out connection with others (friends/family/other pregnant women). In this context, the isolation and loss of social contact during pregnancy was experienced as an acute loss.

Maternity care changes were a major concern for most of our participants. While recognising that midwives were doing their best in a difficult situation, many women had experienced not only a loss of care, but a lack of communication about changes to their healthcare, with reports that midwives have been ‘hard to get hold of’. The loss was particularly acute for women in their first pregnancy, who did not know what they were missing out on.

While some women felt well supported by their midwives, many reported cancelled appointments and classes, face-to-face appointments feeling rushed and stressed and feeling unable to ask questions or share positive emotions. Telephone appointments felt less personal, more removed. Women were particularly troubled that partners were not able to come to the scan appointments, experiencing this as an acute loss, with one worrying about the impact on partner-child bonding. They also felt acutely the loss of antenatal classes – for the important information they were missing out on and the chance to meet other pregnant women. Women wanted ways of replacing these losses, with some mentioning paying for digital antenatal classes, while others simply had no access.

Many women were highly worried about what the birth would be like, and whether their partner could be with them. These concerns about maternity care were stronger than concerns about Covid-19 itself for many of the women in our sample.

#### What information did women want?

Participants expressed a need for more time, support and reassurance from midwives. Women wanted to know more information about their Covid-19 related risk during pregnancy (e.g. why were they in the vulnerable category, what relative risk was associated with each trimester, what is the evidence), their personal risk factors (e.g. comorbidities, ethnicity), clarity on aspects of the guidelines and clarity on changes to their maternity care. Women wanted written information from credible sources, with clear messages presented in an accessible format. Some women reported a desire for lots of detail, while some were experiencing information overload.

### Phase 2: output development

Consultation with HPIG confirmed the authors’ plans to approach midwives with initial findings to gain their input with producing resources to support pregnant women. Midwives were provided with a summary report to guide the consultation process. Midwives agreed that an infographic or brief video would be acceptable for midwives to share, though recognised that as information was changing rapidly, video or written materials could become out-of-date quickly, recommending links to up-to-date information sources. They also recommended producing printable, as well as online, resources to share with women without smartphones/internet access, or for those with limited English who could then access help to read them. The midwives agreed that a more detailed report aimed at midwives would be useful to understand the context of the materials produced.

As a result, we distilled women’s main concerns from our findings to enable us to produce materials that could be shared with pregnant women. The aim was to help answer key questions, direct women to credible, up-to-date information resources and to facilitate conversations with midwives, recognising that communication had suffered due to the pandemic impact. The government, NHS and RCOG websites [4, 13, 16] informed the content, and were included as clickable links for women to access as these were trustworthy information sources that were regularly updated as guidance evolved. We took the following main headings based on our rapid analysis and initial report as a basis for an infographic resource and added summary information for each: *Why am I clinically vulnerable? What is my risk? Should I go to work? Seeing family and friends? Exercise and going shopping? Are there any antenatal classes? What about my antenatal care? What will happen at the birth?*

**Fig. 1:**
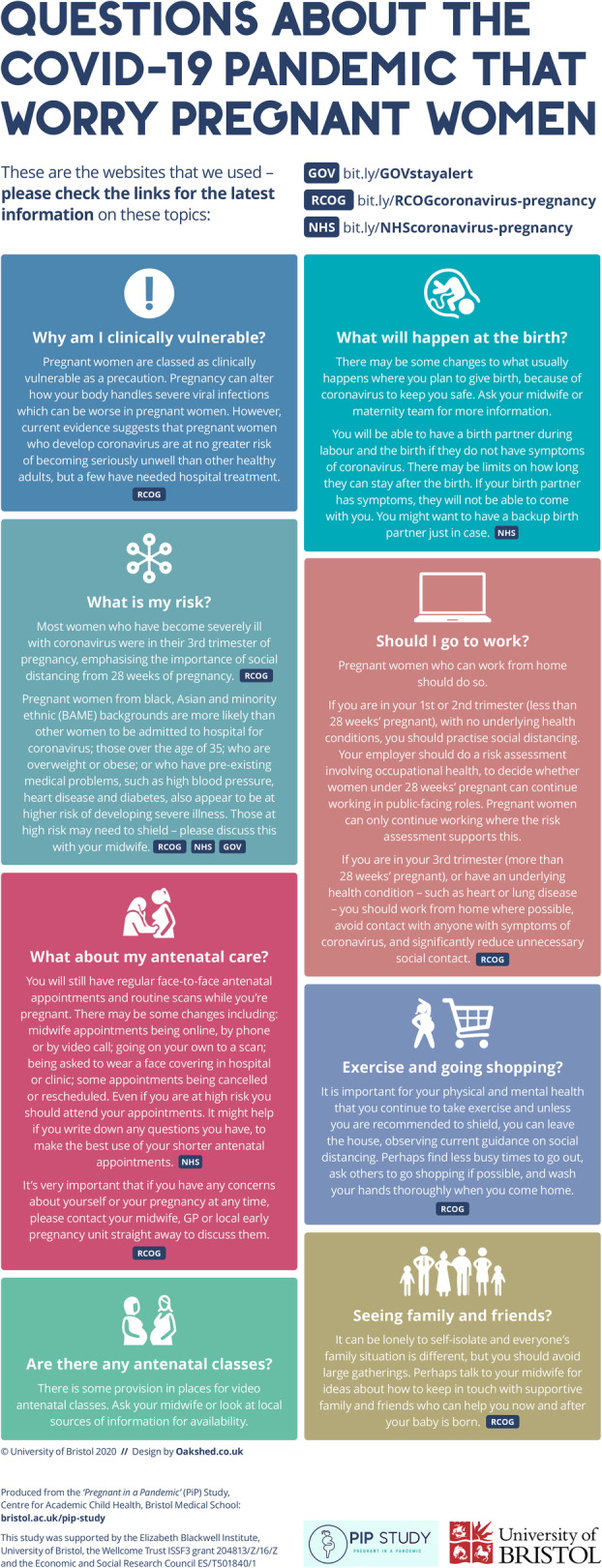
Infographic produced for women pregnant during the Covid-19 pandemic (version for sharing online)

Working iteratively with a graphic designer and consultation with two midwives and two interview participants to refine the content, design and format, we produced an online sharable PDF infographic– [17] see Fig. 1, as well as a printable leaflet and poster version and a moving Graphics Interchange Format (GIF) image of the main questions that linked to the online infographic. We also adapted our initial summary into a report for midwives [18] (see Additional file 4) to give more context and to communicate the findings that midwives may be able to act on or help with. We circulated the infographic versions and midwife report via email to the maternity services at two local NHS trusts as well as providing printed posters for them to display, gaining feedback that these were well received and were shared across teams. We shared the infographic with study participants and the women who had expressed an interest in taking part and shared the GIF and link to the online infographic via social media (Twitter and Facebook) and made these available online [19]. Analytics indicated that since sharing the infographic in July 2020 up to the time of writing (10/05/2021), the Twitter post had gained 3376 views and 751 ‘clicks’ and the study website had gained 352 page views from 332 users (254 in the UK, 39 in the Philippines, 20 in Poland and 20 in Finland) leading to 78 downloads of the infographic.

## Discussion

### Summary of findings in the context of literature

This study is novel in exploring social distancing behaviour in relation to the Covid-19 pandemic in pregnant women, and is the first to apply the COM-B model [5] to assess the determinants of this behaviour in this group. We found that women were adhering well to the social distancing guidance, with many going beyond the recommendations to remain safe. Facilitators of social distancing behaviour were women’s perceived ability to adhere to guidelines (psychological capability), strong conscious desire to stay safe and intentions to continue to comply to mitigate risk (reflexive motivation) with fear as a key driver (automatic motivation). Prevailing strong social attitudes and support helped pregnant women enact social distancing (social opportunity), while work support for furlough/home working and home environmental resources, such as access to a garden and nearby open space were reported as helpful (physical opportunity). There was a lack of clarity around the ‘at risk’ category and what it meant which led some to interpret the guidelines to mean they must shield completely, as well as a lack of clarity around relative risk by trimester (psychological capability). This needed to be addressed to help women understand and adhere to what was expected of them, and was included in the infographic we produced (Fig. 1). The strong reflexive motivation to adhere to the guidance and avoid risk in this group is consistent with findings that reflexive motivation was driving Covid-19-related hygiene behaviour in a general UK population [8].

Women experienced a burden of decision-making, balancing relative risks of exposure to Covid-19 against their needs (e.g. getting fresh air/exercise/attending maternity appointments/support needs around labour). A similar quandary existed in grey areas around the guidance; women wanted to adhere to the rules though uncertainty led to risk-based decisions (e.g. whether to go to work/ shared parenting management/ gaining essential items for the baby). This mirrors recent research showing people are experiencing psychological conflict “between the urge to stay safe and the desire to maintain a normal, pleasurable life” [20], though for pregnant women, the conflict is more between an urge to stay safe (likely to be stronger than in the general population) and meeting *essential needs* rather than pleasure, which is a more severe predicament.

Several factors made social distancing more challenging. These included lack of access to outdoor space, being unable to access online shopping delivery slots and living alone. Pregnant women in our sample tended to respond by further restricting their lives and suffering the consequences – largely negative mental health effects, which was a cross-cutting theme in our interviews. This adds to the burgeoning literature showing the impact of the pandemic and related policies on the mental health of pregnant women and new mothers [21–23].

Beyond the COM-B model, women spontaneously expressed that the loss of maternity care and their anxieties about the birth were their most pressing concerns. Previous studies have reported that pregnant women need clear and comprehensive information about the birth and postnatal care which should be accessible at any stage of pregnancy [24]. Our study has highlighted that the pandemic has exacerbated the lack of information being given during pregnancy and women’s need for reassurance about what will happen. UK research [25] confirms that maternity services have been modified substantially in response to the pandemic with major reduction of services and shifts to remote methods, changes to screening pathways and birth arrangements, calling for more research to understand the impact of these. Concern has been raised about the drastic changes to maternity services to mitigate viral transmission and the resultant reduced capacity of care causing moral injury and impacting the mental health of maternity staff [26]. European research [27] also highlights the rapid changes to maternity care and calls for research to understand its impact on pregnant women. Our study adds new information about the impact on women of changes to their maternity care, as well as trying to address this issue by providing evidence-based resources to facilitate midwife-patient conversations and to address women’s key concerns.

Our study adds to the growing literature on the application of infographics in healthcare and midwifery designed to aid clinician-patient communication [28–30]. The women in our study described Covid-19-related ‘information overload’ as well as lack of clarity on key elements of the guidance and maternity care, and the infographic was designed to address these. Valuable further research could formally explore infographic acceptability and whether the aims to clarify guidance and enhance midwife-patient communication were achieved, as well as exploring what could enhance the uptake and outcomes of such information initiatives in healthcare.

### Strengths and limitations

A strength of our study was that it led to practical outputs (infographic and midwife report), developed iteratively with stakeholder input. The outputs were designed to address women’s main concerns identified in our data (clarifying the guidance, addressing risk, maternity care changes, enabling planning and support) and to facilitate conversations between midwives and patients. These gained positive feedback when shared with local maternity services. Other strengths include the diverse sample, robust data collection and analysis procedures and grounding in behaviour change theory (COM-B). This was a rigorous approach to explore the many determinants of social distancing behaviours in pregnant women in the pandemic context, with the COM-B model providing a framework for the themes identified. During data collection and analysis, we deliberately allowed flexibility so that the findings were not constrained by the COM-B model and were therefore able to identify pertinent data-driven themes (e.g. impact on mental health, concerns about loss of maternity care). We had a robust, systematic process for analysis, following framework analysis methods; all interviews were double coded plus codes and themes were checked during regular consultation with the wider team, which included experienced qualitative researchers and health psychologists.

Work-related comments within interview data suggested that the sample may have had an overrepresentation of university employees which is a potential limitation. We did not capture occupation on the sampling form, and doing so would have allowed us to sample for a diverse employment range, though several different occupations were mentioned within interviews and we had a good demographic range. The sample was self-selecting, so it is possible that the types of individuals keen to participate in a Covid-19 research study may be more engaged or keen to adhere to guidelines than the population at large. We reached saturation on the main themes, though we may not have reached saturation for the range of issues experienced by sub-groups of participants, e.g. those with a chronic health condition or different ethnicities. Understanding the experiences of pregnant women in these more vulnerable categories would be worth exploring further.

## Conclusion (implications for policy and practice)

The confusion of what being ‘at risk’ meant and the varying interpretation of what was expected indicated that there needs to be greater clarity around the categories and guidance. Clarity of policy is especially important given the negative mental health effects of isolation, the extra challenges when pregnant, the psychological burden of risk-related decision-making and potential loss of employment in favour of safety. The loss of maternity care is a major concern for women (and the health care professionals that care for them). When combined with the negative mental health impacts of the pandemic and social isolation, there is likely to be a growing area of unmet health needs which will need to be addressed in future – both around the physical health of the mother and baby during pregnancy and in longer-term maternal mental health. While a *Lancet* paper states that “political leaders must enact quarantine and social distancing policies that do not bias against any population group” [31], it seems pregnant women are disproportionately affected by social distancing policy, and more resources need to be employed to protect the health and needs of this vulnerable group.

## Supplementary Information

**Additional file 1.** Expression of interest form. Form for prospective participants to fill in prior to selection for interview

**Additional file 2.** Topic guide. Questions to guide semi-structured interviews

**Additional file 3.** Thematic analysis according to the COM-B model (as Table 3) with supporting quotations. Table of themes from full analysis including supporting quotations

**Additional file 4.** Pregnant in a Pandemic: Summary report for midwives. Report from initial rapid analysis, produced for maternity staff to accompany dissemination of infographic to two local maternity teams.

## Data Availability

The datasets used and analysed during the current study are available from the corresponding author on reasonable request.

## Abbreviations

BAME: Black, Asian and minority ethnic
COM-B: Capability, Opportunity, Motivation and Behaviour
GP: General Practitioner
HPIG: Health Psychology and Interventions Group
IMD: Index of Multiple Deprivation
NHS: National Health Service
REDCap: Research Electronic Data capture
RCOG: Royal College of Obstetricians and Gynaecologists
UK: United Kingdom

## References

[1] World Health Organization. Statement on the second meeting of the International Health Regulations. Emergency Committee regarding the outbreak of novel coronavirus (2019-nCoV) 2020 [updated 30/01/2020. 2005. Available from: https://www.who.int/news/item/30-01-2020-statement-on-the-second-meeting-of-the-international-health-regulations-(2005)-emergency-committee-regarding-the-outbreak-of-novel-coronavirus-(2019-ncov).

[2] World Health Organization. WHO Director-General's opening remarks at the media briefing on COVID-19-11 March 2020. 2020.

[3] Hsiang S, Allen D, Annan-Phan S, Bell K, Bolliger I, Chong T, Druckenmiller H, Huang LY, Hultgren A, Krasovich E, Lau P, Lee J, Rolf E, Tseng J, Wu T (2020). The effect of large-scale anti-contagion policies on the COVID-19 pandemic. Nature.

[4] Gov.UK. Coronavirus: stay at home, protect the NHS, save lives 2020 [updated 15/04/2020. Available from: https://www.gov.uk/government/publications/coronavirus-covid-19-information-leaflet/coronavirus-stay-at-home-protect-the-nhs-save-lives-web-version.

[5] Michie S, Van Stralen MM, West R (2011). The behaviour change wheel: a new method for characterising and designing behaviour change interventions. Implement Sci.

[6] Chater AM, Arden M, Armitage C, Byrne-Davis L, Chadwick P, Drury J, et al., editors. Behavioural science and disease prevention: psychological guidance2020: British Psychological Society.

[7] Ellis K, Pears S, Sutton S (2019). Behavioural analysis of postnatal physical activity in the UK according to the COM-B model: a multi-methods study. BMJ Open.

[8] Gibson Miller J, Hartman TK, Levita L, Martinez AP, Mason L, McBride O, McKay R, Murphy J, Shevlin M, Stocks TVA, Bennett KM, Bentall RP (2020). Capability, opportunity, and motivation to enact hygienic practices in the early stages of the COVID-19 outbreak in the United Kingdom. Br J Health Psychol.

[9] Centre for Academic Child Health UoB. Pregnant in a Pandemic: The PiP Study 2020 [Available from: http://www.bristol.ac.uk/academic-child-health/research/research/maternal-health/pip-study/.

[10] Harris PA, Taylor R, Thielke R, Payne J, Gonzalez N, Conde JG (2009). Research electronic data capture (REDCap)—a metadata-driven methodology and workflow process for providing translational research informatics support. J Biomed Inform.

[11] Shaw RL, Bishop FL, Horwood J, Chilcot J, Arden MA (2019). Enhancing the quality and transparency of qualitative research methods in health psychology. Br J Health Psychol.

[12] Public Health England. Guidance on social distancing for everyone in the UK: Gov.uk; 2020 [Available from: https://www.gov.uk/government/publications/covid-19-guidance-on-social-distancing-and-for-vulnerable-people/guidance-on-social-distancing-for-everyone-in-the-uk-and-protecting-older-people-and-vulnerable-adults.

[13] Royal College of Obstetricians and Gynaecologists. Coronavirus infection and pregnancy 2020 [Available from: https://www.rcog.org.uk/en/guidelines-research-services/guidelines/coronavirus-pregnancy/covid-19-virus-infection-and-pregnancy/.

[14] Gale NK, Heath G, Cameron E, Rashid S, Redwood S (2013). Using the framework method for the analysis of qualitative data in multi-disciplinary health research. BMC Med Res Methodol.

[15] University of Bristol. Health Psychology and Interventions Group (HPIG) 2020 [Available from: http://www.bristol.ac.uk/population-health-sciences/research/groups/hpig/.

[16] NHS. Pregnancy and coronavirus 2020 [updated 16 October 2020. Available from: https://www.nhs.uk/conditions/coronavirus-covid-19/people-at-higher-risk/pregnancy-and-coronavirus/.

[17] Pregnant in a Pandemic (Pip) Study team. QUESTIONS ABOUT THE COVID-19 PANDEMIC THAT WORRY PREGNANT WOMEN University of Bristol2020 [Available from: http://www.bristol.ac.uk/media-library/sites/ccah/documents/PDF-pregnant-in-a-pandemic-infographic-uob.pdf.

[18] Anderson E, Brigden A, Davies A, Shepherd E, Ingram J. Pregnant in a Pandemic: Summary report for midwives. unpublished (shared via email): University of Bristol; 2020.

[19] Anderson E. Pregnant in a Pandemic: The PiP Study: University of Bristol; 2020 [updated 17/09/2020. Available from: www.bristol.ac.uk/pip-study.

[20] Bacon AM, Corr PJ (2020). Coronavirus (COVID-19) in the United Kingdom: a personality-based perspective on concerns and intention to self-isolate. Br J Health Psychol.

[21] Dib S, Rougeaux E, Vázquez-Vázquez A, Wells JC, Fewtrell M. The impact of the COVID-19 lockdown on maternal mental health and coping in the UK: Data from the COVID-19 New Mum Study. medRxiv. 2020. 10.1002/ijgo.13397 PMC9087547 32979272

[22] Singh P, Goyal M, Singh K, Misra S. COVID-19 and pregnancy: a review. Annals of the National Academy of Medical Sciences (India) 2020.

[23] Davenport MH, Meyer S, Meah VL, Strynadka MC, Khurana R (2020). Moms are not ok: COVID-19 and maternal mental health. Frontiers in Global Women's Health.

[24] McLeish J, Harvey M, Redshaw M, Alderdice F (2020). “Reassurance that you're doing okay, or guidance if you're not”: a qualitative descriptive study of pregnant first time mothers’ expectations and information needs about postnatal care in England. Midwifery.

[25] Jardine J, Relph S, Magee LA, von Dadelszen P, Morris E, Ross-Davie M, et al.. Maternity services in the UK during the COVID-19 pandemic: a national survey of modifications to standard care. BJOG: An International Journal of Obstetrics & Gynaecology. 2020.

[26] Horsch A, Lalor J, Downe S. Moral and mental health challenges faced by maternity staff during the COVID-19 pandemic. Psychological Trauma: Theory, Research, Practice, and Policy. 2020. 10.1037/tra0000629 32478557

[27] Coxon K, Turienzo CF, Kweekel L, Goodarzi B, Brigante L, Simon A, Lanau MM (2020). The impact of the coronavirus (COVID-19) pandemic on maternity care in Europe. Midwifery.

[28] McCrorie A, Donnelly C, McGlade K (2016). Infographics: healthcare communication for the digital age. Ulster Med J.

[29] Siricharoen WV, Siricharoen N (2018). Infographic utility in accelerating better health communication. Mobile Networks Appl.

[30] Smith R, Reid H, Matthews A, Calderwood C, Knight M, Foster C (2018). Infographic: physical activity for pregnant women. Br J Sports Med.

[31] Lewnard JA, Lo NC (2020). Scientific and ethical basis for social-distancing interventions against COVID-19. Lancet Infect Dis.

