# Supplementary material for: Pregnant women’s experiences of social distancing behavioural guidelines during the Covid-19 pandemic ‘lockdown’ in the UK, a qualitative interview study
Source: BMC Public Health. 2021 Jun 23;21:1202. doi: 10.1186/s12889-021-11202-z (PMC8221098; doi:10.1186/s12889-021-11202-z)
Supplement: Supplementary file 1 — Additional file 1. Expression of interest form. Form for prospective participants to fill in prior to selection for interview [file 12889_2021_11202_MOESM1_ESM.docx]

### Expression of interest form including demographic questions

**Pregnant in a Pandemic: The PiP Study Consent to contact form**

We need to ask a little bit about yourself before we book in a time to talk. It is important for us to know a few general details to ensure we talk to a range of different people within our research project.

Are you currently pregnant?

- Yes
- No/ not as far as I know

How many weeks pregnant are you?

- 1-40 [Dropdown answer selection of all integers]

Current age (in years)? ___

How would you describe your ethnicity?

- White

(if selected, specify from: British; Irish; Gypsy or Irish traveller; any other white – please write in)

- Mixed/multiple ethnic groups

(if selected, specify from: White and black Caribbean; White and Black African; White and Asian; Any other mixed/multiple ethnic background – please write in)

- Asian/Asian British

(if selected, specify from: Indian; Pakistani; Bangladeshi; Chinese; Any other Asian background – please write in)

- Black /African/ Caribbean/Black British

(if selected, specify from: African; Caribbean; Any other Black/African/Caribbean background – please write in)

- Other ethnic group

(if selected, specify from: Arab; Any other ethnic group – please write in)

- Prefer not to say

Do you have any medical/ nursing training?

- Yes
- No

Name ____________________

Email ____________________

Telephone number ____________________

Full post code ____________________

**Thank you for filling in this form**. Please note that we may not be able to interview everyone who fills in a form, though if you are interested there may be opportunities to take part in other relevant research. We are very careful with the information you provide, and we only contact you with your consent.

**Would you be happy to be emailed about other relevant research opportunities in future**(with no obligation to take part/ respond)**?**

- Yes
- No
