# Supplementary material for: Pregnant women’s experiences of social distancing behavioural guidelines during the Covid-19 pandemic ‘lockdown’ in the UK, a qualitative interview study
Source: BMC Public Health. 2021 Jun 23;21:1202. doi: 10.1186/s12889-021-11202-z (PMC8221098; doi:10.1186/s12889-021-11202-z)
Supplement: Supplementary file 2 — Additional file 2. Topic guide. Questions to guide semi-structured interviews [file 12889_2021_11202_MOESM2_ESM.docx]

### Main topic guide

| 1. **Quick check - do you have children already?** Y/N |
| --- |
| 1. **Do you think you or anyone close to you has had Covid-19 (coronavirus)?** |
| 1. **What do you know about social distancing to prevent you catching coronavirus during your pregnancy?** (psychological capability)   Prompts:   - What kinds of things do you think you should or should not do? - What is the advice you have been given or read about social distancing?   *Only if they report having no idea at all (v unlikely):*   - *prompt with current social distancing recommendations – see end of this doc** |
| 1. **Are you currently following all, some or none of the recommended advice about social distancing?**  (current behaviour)  - What are you doing differently? - If some/none: which bits are you following or not following? |
| 1. **Do you feel confident you know *how* to do all the things that are recommended?** (psychological capability) |
| 1. **Is there anything that prevents you from doing the recommended things on a practical level?** (physical capability) |
| 1. **Do you intend to follow all the recommended advice about social distancing?** (intention) |
| 1. **What do you think will happen if you do or do not follow the recommended behaviours?** (reflective motivation)  - e.g. what are the benefits and drawbacks of doing them or not doing them? - do you think those things that might happen are likely to happen for someone like you? |
| 1. **Have you established any habits or routines that are helping you to follow the recommended behaviours?** (automatic motivation) |
| 1. **Is there anything in your current situation that is helping you or preventing you from doing what is recommended?** (social opportunity/ physical opportunity)  - e.g. for example, your living circumstances, your job |
| Wait for answer, then if it hasn’t come up already, prompt   - Are you currently working? If yes: - What is your job and are you working from home? - Do you think your job has any impact on your ability to follow social distancing? |
| 1. **What do the people around you think about following the recommendations in pregnancy, for example your partner or family?** (social opportunity) |
| 1. **Where do you get information about managing potential risk of Covid/Coronavirus during pregnancy?** (extra question/ related to psychological capability) |
| 1. **What information would you find helpful and who would you like to hear it from?** |
| 1. **Are there some other comments you would like to make on what we have talked about today?** |

** Guidance published on the Public Health England website at the time of interview:*

***What is social distancing?*** *Social distancing measures are steps you can take to reduce social interaction between people. This will help reduce the transmission of coronavirus (COVID-19). They are to:*

1. *Avoid contact with someone who is displaying symptoms of coronavirus (COVID-19). These symptoms include high temperature and/or new and continuous cough*
2. *Avoid non-essential use of public transport when possible*
3. *Work from home, where possible. Your employer should support you to do this. Please refer to*[*employer guidance*](https://www.gov.uk/government/publications/guidance-to-employers-and-businesses-about-covid-19)*for more information*
4. *Avoid large and small gatherings in public spaces, noting that pubs, restaurants, leisure centres and similar venues are currently shut as infections spread easily in closed spaces where people gather together.*
5. *Avoid gatherings with friends and family. Keep in touch using remote technology such as phone, internet, and social media*
6. *Use telephone or online services to contact your GP or other essential services*
