# Supplementary material for: Pregnant women’s experiences of social distancing behavioural guidelines during the Covid-19 pandemic ‘lockdown’ in the UK, a qualitative interview study
Source: BMC Public Health. 2021 Jun 23;21:1202. doi: 10.1186/s12889-021-11202-z (PMC8221098; doi:10.1186/s12889-021-11202-z)
Supplement: Supplementary file 3 — Additional file 3. Thematic analysis according to the COM-B model (as Table 3) with supporting quotations. Table of themes from full analysis including supporting quotations [file 12889_2021_11202_MOESM3_ESM.docx]

Thematic analysis according to the COM-B model (as table 4) with supporting quotations

| **COM-B category** | | **Themes** | **Participant Quotes** |
| --- | --- | --- | --- |
| ***BEHAVIOUR*** | | | |
| Social distancing (in accordance with guidelines) | Adhering | | ID46: “I have just been following the guidelines of staying at home unless needed to go out. I am still going out to do the food shopping stuff but obviously keeping a couple of metres apart. I have not completely isolated myself, but yeah just staying at home really, doing a bit of exercise outside but not much.”  ID60: “We will go for a walk, and that’s it, that really is it. We don’t see anybody, we do our food shop once a week, he will go in, I won’t even go to the shops anymore.”  ID85: “I only go out of the house once a day to walk the dog, so social distancing from people that I come across, so just going across to the other side of the road if I see someone approaching me.”  ID03: “since the lockdown came in I haven’t come into close contact with anyone apart from my husband. So yeah we have been staying indoors, going out once a day.”  ID24: “I have been only going out for one walk a day, and only at times that are really quiet. I haven’t been going to the supermarket at all, we have just been getting deliveries or my partner has been going sometimes but not often really, and poor [name] our toddler because life has become very boring we’re only doing short walks, and when we are out staying two metres away from everybody.” |
|  | More extreme | | ID53: “I have been personally isolating since the 8th of March, which was well before the UK went into lockdown, because of my background I knew what was coming and I didn’t want to expose myself to any risk.”  ID79: “I have been isolating since 17th of March, so I haven’t been out of my house at all. I haven’t been to the shops for God knows how long, the only time I have been out really is to go to my midwife appointments and my scans, and then as soon as I get home from my appointments I take off all my clothes downstairs and I literally go up in the shower and have a shower and wash myself. Although I have spoken to my midwife about it and they say I don’t need to do that really, but for me and my own piece of mind that’s what I would rather do just so I know that I am clean.”  ID03: “I noticed that the status for pregnant women had changed to vulnerable, it was the week before or something I think, so we started self-isolating before full lockdown came in.”  ID19: “I have just not been going out at all, we’ve been getting the shopping ordered in […] we’ve got no one who can really risk going out to shops.” |
|  | Slight deviations | | ID25: “Probably some because I often go out more than once… not often but go out more than once a day potentially to exercise to take my two-year-old out of the house. So in terms of am I following it strictly no, because we will go out sometimes for two walks a day instead of one. In other senses I feel as far as my understanding of it is that we are adhering to social distancing rules.”  ID13: “we’ve had many conversations about interpretation, so something that we have decided on is because I’m a very active person and it’s been quite hard for me not to be so active. So we have been driving and going for walks, or making sure that I am out and about and going for longer walks than others might be because that’s probably as important to me in terms of my pregnancy actually to have that balance. So that’s been the main thing. We have gone to my partner’s family house just the once to go and collect some stuff from them, but we kept away from the family, we were quite careful about that”  ID21: “The only thing that we have done is we have actually moved in with our parents now, so they isolated for about two weeks and we isolated for a week and then we moved in together, some more support.” |
| ***CAPABLITY – The individual’s physical and psychological capability to engage in the behaviour(s)*** | | | |
| **Psychological capability**  (understanding/ mental processes) | | Knowledge and understanding of guidance around social distancing behaviours | ID45: “So to only leave the house if I need to apart from for daily exercise, and then when I’m on daily exercise keep two metres away from people, and then I can collect medicines if I need to or essential items like food, and working from home if I can, which I can, so that’s fine. So I think that’s all. Not having people round the house and staying in.”  ID18: “I am very careful about reading stuff on reputable sources sort of thing”  ID24: “I think as it is right now it’s been quite clear.”  ID30: “I just find them a bit vague when it comes specifically to pregnancy, I don’t think the messaging is really clear. So it suggests that we’re vulnerable but then we’re not in the really vulnerable group, and it’s a bit grey, so I am not quite sure once I tell me employers I am pregnant how I will be treated, I don’t know if they will say I need to stay at home and shield.”  ID62: “…pregnant are in the vulnerable category made it seem like everyone who is pregnant should just be very strict and stay indoors and not see anyone.”  IID01: “from what I have seen on the news or the NHS guidelines it has been advised that pregnant women should social distance for 12 weeks, self-isolate, so I live with mum and dad and so we’ve not really been going out at all”  ID09: “I don’t know, even though I am pregnant and I am classed as high risk category I don’t feel like I am a risk, and I don’t feel scared about it. I don’t know, maybe I should be more, but I think if it was really explained a bit more that no actually you have… there’s a very high risk you could have a miscarriage if you got it then I would be really… I don’t really understand why. So I think if I understood it a bit more I might maybe take it a bit more seriously.” |
|  |  | Confidence in ability to enact social distancing behaviours | ID40: “Yeah, I feel confident on how to do it”  ID24: “I think maybe there’s some nice time that we have had with our toddler and with my partner which we wouldn’t normally have had this much time together before the baby comes, so there’s definitely trying to see some nice things with it.”  ID19: “I think as time goes on it will get more difficult just in terms of mental health”  ID05: “there are times where I just sit in the house and I cry and I am just like I can’t do this, I just need to get out and see a human being” |
| **Physical capability** | | Physical capability had little impact on social distancing behaviour | ID85: “I think I am pretty good with the social distancing”  ID31: “she might want to run over and say hello […]But it’s a bit difficult, and also with the stage of pregnancy I am at it’s a bit more difficult to physically pick her up” |
| ***OPPORTUNITY – Environmental factors influencing the behaviour(s)*** | | | |
| **Social opportunity** | | Social norms to comply with social distancing | ID05: “Oh no they [friends and family] are super strict with it, they are like no you’ve got to follow it, you’re pregnant. It’s all about keeping you and that baby safe”  ID45: “my husband he is quite strict on me following… not in a mean way but he’s very keen on, because I said, “I will just do the shopping it doesn’t matter,” kind of thing, and he is like, “No definitely not, I’ll do it,”  ID06: “At the start of the situation I did get a number of comments from colleagues about me being in work”  ID17: “Everyone else has been following the rules as well”  ID53: “they [family] are very supportive and they are all very strictly adhering the guidelines as well. Regarding my friends I found it a bit distressing that some friends have just gone off on one with conspiracy theories and why do we need lockdown, we don’t need lockdown, and I have lost some friends over it which has been really upsetting” |
|  |  | Household composition impacts on ability to enact social distancing | ID37: “living with my husband means that he can go out and do the food shops, so that’s super helpful. I think I would be really reliant on neighbours or friends if I didn’t have that”  ID19: “Having a family who are all isolating as well is really helpful, I don’t know how hard it must be for people who have family members who are key workers who aren’t able to socially distance properly.”  ID25: “I suppose I still have to take my son to nursery which is difficult to get round because my husband is also a key worker but he is working from home so he can’t be free to do that, so I suppose I am going into another environment with him daily before and after work. But other than that I feel like we’re managing okay.”  ID05: “There’s nothing that’s preventing me from doing what is recommended, but it is a lot harder, because I live on my own” (ID05) |
|  |  | Social distancing compromised by strangers in public spaces | ID32:“they can’t see that I am pregnant, but then they do see me veering across the grass with a heavy pram, and yet they don’t move out the way, and I think well you can’t just keep driving a woman with a pram across the grass because you are not moving, seems a bit inconsiderate sometimes”  ID03: “sometimes I feel like having a big sign saying ‘I am pregnant stay away’” (ID03)  ID83: “Some people just don’t seem very accommodating, they just walk right up to you when you’re on the sidewalk, they could wait. […] if I’m completely in control of my environment I can do everything, but sometimes it’s other people.” (ID83) |
| **Physical opportunity** | | Impacts of home environment and resources | ID40: “it’s difficult because we haven’t got a garden, so even if you wanted to go out and do your daily exercise in your garden or just go out and have some fresh air I couldn’t, and it makes it more difficult again because I live on a high street, I don’t want everyone walking past me”  ID24: “I do think having a garden makes such a big difference, I can really imagine if I lived in a flat I would be much more likely to go out more than once a day”  ID05: “I live in a tower block, so that’s why I was saying the only fresh air if get is when I go down to the bins.”  ID39: “we’re very lucky we’ve got a rowing machine so I have been doing rowing, and we’ve got some kettle bells, because I do a lot of stuff at the gym normally so I would go a bit mad if I didn’t get to do that.”  ID62: “The things that are helping me are just being lucky enough to have a car so I can just sit in a car on my own and drive myself to work without having to worry about public transport.”  ID54: “technology generally has helped loads, just being able to see friends but on a screen or see colleagues but actually see their faces has made a massive difference to communication” |
|  |  | Work environment/ ability to work from home | ID46: “I feel really lucky that I am able to carry on working online, so that’s really good.”  ID03: “it has been quite helpful being able to work from home, just health point of view, so there are some upsides to it”  ID32: “I think they are prepared for me to be a protected member of staff and work at home until the end.”  ID30: “I am a little bit nervous about what’s going to happen next in terms of if we have to go back whether or not it will mean that there’s more chance of catching it, I am a little bit worried about that because I can’t control it then.”  ID25: “I was able to suggest that perhaps I can still go to work, I minimise my patient facing contact, that sort of thing, and work from small offices rather than in a large room with all the clinicians […] there was I would suggest unspoken pressure to remain in work.”  ID62: “I think it would be really bad for me mentally if I couldn’t come in, and financially because I’m a freelance, and so if I don’t work I don’t get paid. So she was like, “If you feel well and you feel happy and safe and okay to do that then I am happy.” So I just carried on, and she just said, “Flag up if you don’t feel safe, flag up if you don’t feel well or you want to just go home, just let me know.” So far I have just felt fine so I have just carried on.”  ID53: “if I am pressured to return to the office while the virus is still at large I will have to take sick leave because I can’t take that risk”  ID60: “there’s 1,000 people in my office, some young, some old, some of them have been travelling etc., I work front of house, I just decided that I wanted to finish up because I have to get public transport into work[…]I was going to have to be put down onto sick pay which I think was £94 a week or something, but then the CEO of [insurance] she found out that I was pregnant and I was taking time off and they send in the end no I want her on full pay. Because I did worry about the pay to begin with but they sorted it out and put me on full pay, which is a bonus in itself.” |
|  |  | Shopping for essentials including preparation for the baby | ID17: “The shopping, it’s just I am trying my best to get a delivery slot just because then I haven’t got to go out, I can stay home safe, but needs must and you’ve got to eat haven’t you? So that’s the only thing.”  ID01: “you have to rely on someone else maybe pick it up and drop it at your door. […] I am lucky that there are people around that can get us stuff.”  ID46: “it’s the highlight of the week being able to do the food shop at the moment”  ID53: “I have had to go without food because I wasn’t put on the risk list I have had long gaps between supermarket deliveries […] and nobody to help me, and that’s meant that I haven’t been able to get fresh food. […] So I have been trying to compensate for that by growing my own food.”  ID14: “The only other thing is the difficulty in getting baby things really, because you try and order stuff online and a lot of things have been out of stock, and then you can’t go to the shops and buy these things.”  ID13: “my family is going to give me, friends and family for the baby, and so that we don’t have to buy everything new, and it’s those kind of things of so how do we manage that as it gets closer to the due date, and do we do some kind of handover in a car park” |
|  |  | Healthcare appointments | ID32: “The midwife appointments we do mostly from the car, but I have to go in to have my blood pressure checked, and then the worst thing was last week my scan, my husband wasn’t allowed to come in with me so I had to go in to do my first scan on my own which was horrible. But yeah everything has either been done in the car or with masks on and by myself.”  ID39: “going for example into the hospital for my 12-week scan worries me a bit because that’s a pinch-point where there are going to be other people and lots of people”  ID40: “Now everyone has got their masks on and it’s in and out as soon as possible.”  ID85: “for my scan no partners were allowed in, but no I just kept my distance from everyone, in the waiting room we were separated from other pregnant women as well, so no it was all very well signposted and we were made aware of what to do”  ID37: “I think the antenatal appointments are probably the only time when I come into contact with another human being, and some of those like with the scans and stuff it’s been… it’s obviously impossible to be two metres apart, but all the antenatal staff have had all PPE, they have all been completely masked and gloved and things like that.”  ID82: “my husband couldn’t go with me, and I had my mask with me, on me, but they were giving masks on the entrance to the hospital as well, and they ensured that there was social distancing in the waiting area as well.”  ID83: “The only time that I have socialised with people is to go to the appointments, and I don’t know how things will go in the second half, and if there will be any more need to see doctors. So that’s the only… that would be the only reason why I would come into contact with someone”  ID19: **“**getting to the hospital is alright, going in someone else’s car, because we’ve all been isolating, but on the way back I will have been into the hospital and potentially could have picked something up. So on the way back, this might sound like it’s totally overboard, I don’t know, but we’re going to use some plastic sheeting and tape up the back of the car, and I am going to get into the back like a quarantine zone” |
| ***MOTIVATION – Individual internal factors that direct the behaviour(s)*** | | | |
| **Reflexive motivation** | | Motivated to adhere to social distancing guidelines | ID41: “by following the recommend behaviours I am thinking that I am doing the right thing, I am protecting myself and my family and protecting the NHS”  ID46: “the main thing is I want to make sure that the baby is healthy and I’m able to go into hospital and have a safe birth”  ID60: “I definitely will follow it because then it’s not just me to think about, there’s a baby and the poor baby is not going to have an immune system, so yeah we are not risking anything. It’s just not worth it.”  ID39: “I know that you’re a bit immunocompromised or immunosuppressed aren’t you when you’re pregnant, because you’re more likely to get infections and things like that.”  ID53: “I’ve got a high risk pregnancy and various other risk factors so I considered it essential that I didn’t expose myself to the virus.”  ID79: “I definitely don’t want to get it being pregnant and being part of the black ethnic minority group”  ID13: “we’re just really wary of getting it around time of birth” |
|  |  | Establishment of routines to enable social distancing | ID62: “Just staying at home really is the main routine. Cooking more at home, just routines like just not… I don’t know it just feels quite normal now I think, the new routine is just go home and stay in, and it feels quite normal”  ID36: “routine wise I try and get outside everyday if I can, especially as we’ve got a park nearby, for a walk or a run, but I am doing less running these days, and just I find that helps to keep me sane really getting outdoors and exercising. I think planning ahead for shopping really helps, so I have been doing a lot more making shopping lists before I go out rather than just seeing what I find when I get there, and that really helps to reduce the number of times I have to go.”  ID37: “We have quite a strict regime to stop my husband and I killing each other mainly [laughter], so we get up and start work at nine, and then we have lunch during the day, and then we finish about four, and we tend to have a little maybe a walk around the park.”  ID45: “Having structure in the day I think has really helped me, although I haven’t done it so much lately but doing… I do the Joe Wicks thing in the morning, I find that really helpful just to… I don’t know, especially the days when I’m not working I can just get a bit low, deflated, not much to do kind of thing, and no one to see. So it helps to have structure I think. That’s helped a lot, and going out for an afternoon or evening walk, that’s really helped, and speaking to family a lot on Zoom” |
|  |  | Intentions to continue to adhere to guidelines | ID79: “I will follow the rules and procedures, and even if they do say oh yeah it’s still… it’s okay you can go out, until I feel for me mentally I feel that I am safe and I feel that my family is safe then I might want to embark on going out properly and meeting people. Until then I plan on staying in my house.”  ID18: “Yeah, certainly intend to follow the advice as the government are recommending it, and I think they have been making the right decisions so far with things. In fact if anything I am more concerned about things being lifted too quickly”  ID21: “I haven’t read any information or recommendations about a newborn baby, but makes sense to me that you would want to keep them far away from any potential danger, so I imagine we will be carrying on as we are until all the recommendations change, and even then I don’t know if we would change what we’re doing for a while.”  ID46: “at the moment I am feeling like if it were to continue like this and the baby were born I probably would start letting people come and see me if they were all clear, if they definitely didn’t have the virus. So I do think there’s going to be a time where I feel like life needs to start happening, I don’t want to feel like my family are missing out on the first few months of the baby’s life, so I guess that’s something that me and my partner will weigh up once the baby is here.”  ID13: “this is all fine whilst baby is still inside, when baby is out it feels like a whole set of different circumstances for a few reasons, for the fact that we might need more support from friends and family, the fact just want them to meet baby and experience baby when they are young. […] we have been following them now and even more strictly maybe as we get closer to the birth. It’s when baby comes I think… I am really hoping there will be some kind of loosening of the rules a bit, so we get to see people.”  ID37: “we have discussed this, if we’re still in a severe lockdown at the time of mid-August so we would move in with my parents, because they have got a bigger house, because not having anyone to visit with the first few weeks of a new baby I think would be mentally quite detrimental.”  ID54: “I think it’s going to get harder depending on what things look like when the baby arrives, because I may need a backup birth partner, I would probably want that to be my mum, but they’re two hours away, […] So I feel like that’s my big worry, that’s where I think we might reach a breaking point and have to go right we’re making this concession because I don’t know if I can do it, and if I have someone else physically here to help me they are the only circumstances I can think where I would be willing to really risk that.” |
|  |  | Risks and balance of risks to determine behaviour | ID53: “So I have had a midwife appointment over the phone rather than my midwife, and I am having less appointments than I would normally have which is quite worrying, but you have to weigh up the risk of having the appointment and catching the virus, exposing yourself in a high risk environment like a hospital.”  ID13: “I went to a little shop just because I just felt I needed some control over what I was going to eat, so just a small excursion to a small shop. But we feel the risk benefit of going and doing a long walk out in the open or going somewhere quite remote the benefit massively outweighs the risk for me and my mental health.”  ID21: “So when we were having discussions around work it was a decision based on risk”  ID45: “I don’t think I am an at risk group or anything […] So yeah I was just thinking I don’t know if the risk of me going in and exposing myself to loads of… because my midwife clinic is at the GP’s so I would be exposing myself to those ill people [laughs], and I thought that might not be worth the risk just for something that’s quite unlikely anyway.”  ID54: “I’ve got a friend up the road who has got a toddler, it’s really tempting to want to go for a socially distanced walk with them, but I don’t know if that’s safe, and that would make me feel a lot better and give me a big lift, but it feels wrong or it feels like it would be an unnecessary risk, and I would be contributing to a problem.”  ID82: “given that we are going to have a month old child in the house and me post birth as well and immunity system being affected by that as well I am not sure if I will be that keen to send her to school straight away because it may not affect her that much but she may bring something into the household that will affect all of us here. So again that’s something we will have to think about closer to that time.” |
| **Automatic motivation** | | Emotional drivers of social distancing | ID83: “I don’t care what anyone says, I don’t want to get sick, I hate to sound dramatic in case you’re writing down our worst fears, my worst fear is that I lose my husband”  ID79: “I personally I really don’t want to get this, I am even holding back tears now talking to you. No I just I don’t even want to think about what will happen if I don’t follow the procedures and the protocols that they have set in place, because I am really worried about getting it, and especially getting it whilst pregnant”  ID54: “I don’t feel as worried about contracting it in a way, although obviously I don’t want that and that would be awful, I am not a high risk person in terms of my health normally so I don’t feel unduly worried about that, I just feel really worried about the capacity that we have.”  ID45: “if it’s something I could have controlled. If I can’t control it it’s not… that’s just that, but if I could have just not gone out a few times I would have felt really guilty.”  ID13: “I am flitting between a gut feel which is I will be fine, I have got a very low risk pregnancy and generally fit and well, but feeling guilt just for going out on a walk, and that’s not a happy place to be in.” |
|  |  | Automatic behaviours | ID17: “At first I was a bit confused I think, but I think now that I’ve got used to it I think it’s more of a daily thing now if you just… you don’t even think about it anymore.”  ID31: “I think it’s just the same as everybody, if you’re out… if you do have to go somewhere like a hospital or a shop or whatever I think most people are doing that automatically staying away from people” |
| ***Beyond COM-B: cross-cutting themes*** | | | |
| **Isolation, mental health, and loss of maternity care** | | Isolation and mental health impacts | ID41: “I think the isolation side of the lockdown is probably the most difficult bit, which I think I feel perhaps more acutely being pregnant.”  ID46: I think that’s the big thing, it’s like being in lockdown you’re really not able to connect with other people in your situation”  ID13: “pregnancy is meant to be a really joyful lovely time that you get to share with everyone, and that gets taken away  ID83: “I just think I really do miss… I feel like there’s a gap in my life about hearing from other people”  ID13: “my family support networks are far away, they were going to come today, that can’t happen, I don’t know what services are available to me, and I am worried about that. There are probably more and more women who are at risk and feeling isolated in their homes”  ID54: “a few of us do struggle with anxiety, and there’s been a lot of talk about managing anxiety which I am sure would be happening at a low level anyway, but there’s a lot of people trying to manage it by themselves at this point, and none of us have given birth yet […] I don’t really think you can assess that [mental health] properly via the phone unless somebody feels willing to flag that themselves, and often I don’t think you know that’s going on properly until it gets to a crisis point or you are beyond it. I am quite concerned about that element”  ID05: “no one is doing any checking or anything to say how is your mental health, how are you doing, you have asked us to do all of this stuff but what are you doing for us.” |
|  |  | Maternity care – loss of communication | ID40: “they rang up the day before because I didn’t know what was going on with my booking appointment until the day before, because I thought I was just going in and I couldn’t get hold of them to ask if that was still going ahead and things, and the day before the appointment she rang me just to say it’s going to be over the phone, and then I needed to come in the following week for a blood appointment”  ID01: “I haven’t had any updates or any antenatal classes, I am assuming they are not going to be run or go ahead” |
|  |  | Loss of maternity care | ID06: “this is my first time so I don’t know what I should be getting versus what I am getting.”  ID05: “this is my first child, I don’t know what the hell I am doing, so it’s just like all those appointments are really important to me”  ID79: “I just feel like everything has just been snatched away, I can’t enjoy it. We can’t even go and have our scans together”  ID40: “everyone is stressed, and all the services are stretched, you just don’t want to feel like you want to not waste their time, but normally you would be able to go to someone or go to a service like your midwife and ask them and they would have time to speak with you […] but because they are so stretched now you just don’t feel like you want to… everyone bangs on about save the NHS and you don’t feel like you want to go in there asking anything”  ID41: “I think there are other benefits though to having the face to face classes that means I am missing out on meeting other local mothers.”  ID40: “all the classes have been cancelled, and you can’t just go… you feel like you haven’t got time to sit in there and have a chat and ask these questions that you want to ask”  ID31: “birth partners being allowed in hospitals and not being able to stay after even if they are allowed for the birth as result of Covid. […] is one thing I’m am anxious about more so that the risk of catching it.” |
