# Supplementary material for: Pregnant women’s experiences of social distancing behavioural guidelines during the Covid-19 pandemic ‘lockdown’ in the UK, a qualitative interview study
Source: BMC Public Health. 2021 Jun 23;21:1202. doi: 10.1186/s12889-021-11202-z (PMC8221098; doi:10.1186/s12889-021-11202-z)
Supplement: Supplementary file 4 — Additional file 4. Pregnant in a Pandemic: Summary report for midwives. Report from initial rapid analysis, produced for maternity staff to accompany dissemination of infographic to two local maternity teams. [file 12889_2021_11202_MOESM4_ESM.pdf]

redacted logo

PIP STUDY
PREGNANT IN A PANDEMIC

redacted logo

Pregnant in a Pandemic: Summary report for midwives

In the Pregnant in a Pandemic (Pip) Study we interviewed Bristol women (between 24 April–7 May 2020) at all stages of pregnancy about their experiences of social distancing in the Covid-19 pandemic.

Findings: a need for more information and support for pregnant women

Current social distancing behaviours: Women seemed well-informed about the main social distancing messages at the time (keeping at least 2 metres apart if going out, staying home as much as possible, limiting shopping) and were following the guidance to the best of their ability, with some taking it to greater extremes (e.g. washing shopping, completely shielding). While women were generally adhering well to social distancing guidelines, they expressed a need for more support, and had specific concerns around managing the risk of Covid-19 during pregnancy. Below we highlight key issues that were identified from our research.

‘It’s quite a scary time to be pregnant, and I think personally I feel like the more information I have the more reassured I would be’

Stress and uncertainty: Pregnant women have been categorised as “clinically vulnerable” or “at risk” from coronavirus and advised to be particularly vigilant in observing the social distancing advice given to the general population. Together with pandemic-related changes to antenatal care, this has had a range of effects on women, with many feeling anxious, stressed, uncertain and isolated, as well as worried about their partner missing out or not being able to attend important appointments. Women were anxious about the easing of lockdown and many planned to continue with stringent social distancing when it eased.

The importance of midwives in providing information: Women reported having fewer or shorter contacts with their midwife, less face-to-face time and less partner involvement in appointments as services adjusted practices for social distancing, which they experienced as a loss. First time mothers in particular felt they were missing out on antenatal care, time to ask questions, to connect with other pregnant women, and felt a loss of positive emotion about being pregnant. Women were keen to talk through what Covid-19 means for them now, for the duration of the pregnancy, for the birth and for the care of their baby after the birth.

Communicating change: Women wanted clear proactive communication about changes to guidance and to their antenatal care. While some reported that they had good communication from their midwives, others found their midwife “hard to get hold of” or said they “haven’t had any updates or any antenatal classes”.

What women worry about

Below, we present women’s key questions and concerns. While wanting answers, women recognised there were many unknowns, and valued transparency when information was not yet known or was a best guess.

- **WHAT IS THE GUIDANCE? – A NEED FOR CLARITY**

While women generally understood the social distancing guidance, there was uncertainty about some details (e.g. whether to attend appointments, what household members should be doing), and some confusion, for example between the terms ‘self-isolating’ and ‘social distancing’. Women need to know where to access clear, up-to-date guidance, particularly when the advice is changing rapidly.

- **WHAT DOES BEING “CLINICALLY VULNERABLE” or “AT RISK” mean?**

Women wanted to know what this means: How they are different from the general public, and from the group who are fully shielding? Women wanted to know the rationale for this classification; wanting to understand the evidence base around risks, or if this categorisation is precautionary. Some thought pregnant women were classed as clinically vulnerable because pregnancy alters the immune system.

[redacted]

[redacted]

❖ SHIELDING (NOT LEAVING THE HOUSE) AND ITS IMPACTS

Women who were shielding did so because: (i) they interpreted guidelines, and the 'at risk' (clinically vulnerable) category for pregnant women to mean this was necessary; (ii) they chose to be extra cautious due to anxiety, or (iii) they, or a household member was 'high risk' (clinically extremely vulnerable) for other reasons. Particularly for single women, shielding was negatively impacting their mental health ("there are times when I just sit in the house and I cry") and their ability to manage daily tasks, such as obtaining food.

❖ PERSONAL RISK

Women felt the Covid-19 guidance for pregnant women was generic and wanted individualised information, for example, risk implications for different ethnicities and those with chronic or other health conditions.

❖ DOES THE TRIMESTER MAKE A DIFFERENCE?

Women were keen to know if there were different risks associated with Covid-19 during the different trimesters, and the rationale or evidence base for these. They also wanted to know if there was different guidance about what they can and cannot do at the different stages of pregnancy.

❖ CHANGES TO MATERNITY SUPPORT

a) Antenatal classes: For many women, antenatal classes were now unavailable. They were concerned about losing out on both (i) the important information and (ii) the contact with other pregnant women, which was valuable to them. Women wanted ways of replacing these losses, with some mentioning paying for digital antenatal classes, while others simply had no access.

'all the classes have been cancelled, and you can't just go… you feel like you haven't got time to sit in there and have a chat and ask these questions that you want to ask'

---

b) Routine maternity care: Women suffered disruption to routine care, and many experienced a lack of communication from health services, which was unsettling. They were keen to know what was happening with their care in advance of appointments and changes, ideally via proactive contact from the service to inform them about:

- what is the same and what is different in their maternity care
- what they are missing out on
- what to expect when they visit
- what actions clinics are taking to keep them safe during visits
- clear rules about partners attending each type of appointment

c) Partner involvement: Women expressed anxieties and sadness around their partner not being able to attend maternity appointments and scans ("I just feel like everything has just been snatched away, I can't enjoy it. We can't even go and have our scans together"), feeling partners are missing out.

d) Practicalities of visits/ combing appointments: Attending face-to-face appointments was challenging for some women due to difficulties maintaining social distancing and arranging childcare (one woman was arranging a contact to shield for 2 weeks prior to the appointment in order to look after their children). Repeat visits were a source of anxiety, particularly for those with extra health issues. If women needed several face-to-face appointments for physical examinations, bloods, scans and tests, they would like these combined where possible.

e) Time to talk: Women acknowledged that maternity services had to change rapidly and put in protection measures, but appointments now felt rushed. The sense of needing to "protect the NHS" also led to women not wanting to take up NHS time. Losing antenatal classes and having less face-to-face time with midwives meant missing out on time to ask questions (even 'silly questions') and have concerns addressed, as well as losing a sense of connection and the joy of pregnancy. Women felt less connection with their midwife in telephone appointments compared with face-to-face contact.

[redacted] PIP STUDY PREGNANT IN A PANDEMIC [redacted]

'I don't really think you can assess that [mental health] properly via the phone unless somebody feels willing to flag that themselves'

❖ **CONCERN ABOUT OTHER HEALTH CONDITIONS**

a) **Routine/ minor health conditions**: Women described being wary about contacting healthcare services for minor health conditions, not wanting to add pressure to the NHS. There was some uncertainty about whether to raise 'minor' health concerns, or what counted as important or minor.

b) **Chronic/ complex health conditions:** Women with other health conditions felt particularly vulnerable during the pandemic and were keen to have more personalised information such as: what are the risks, what actions should they be taking in terms of social distancing, what appointments should they attend.

❖ **WHAT WILL HAPPEN AT THE BIRTH AND AFTERWARDS?**

a) **Changes to care around the birth:** Women had anxieties about what the birth would be like during the pandemic, especially concerning partners ability to attend and how long they may stay. This was a greater worry for some than catching Covid-19, while other worries arose around impacts on partner-child bonding.

b) **Social distancing during labour:** Women recognised they would be unable to be socially distant from medical staff during labour, while single women would need someone to take them to hospital. Women with other children were concerned about how to manage childcare so that their partner could come to hospital, having to break social distancing guidelines to put support in place. These considerations were a source of anxiety for women, as they were forced to weigh up the social distancing guidance and risks of coronavirus against their support needs during labour.

a) **Covid-19 infection when due:** Women wanted to know how the birth experience would be impacted if they caught Covid-19 at/near their due date, especially whether they would still be able to give birth in hospital, impacts on planned C-sections, and what would happen if their partner was infected.

b) **Medical care after the baby is born**: Women wanted to know what to expect after the birth in terms of midwife and health visitor visits, medical appointments and guidance.

❖ **WORK CONCERNS AND SUPPORT NEEDS**

Some women found working from home beneficial (e.g. time with family, flexible hours) and reported that employers had prioritised pregnant staff for furlough/home-working (aided by being classed as 'at risk'). Others reported pressure to continue working without social distancing/safety measures in place. Women perceived social judgements and guilt about both going and not going to work. With lockdown easing and without clear guidance around return to work for pregnant staff, women were anxious about safety. This was particularly of concern for those with extra risk factors (e.g. comorbid health issues, Black ethnicity) not currently in the 'extremely clinically vulnerable' classification. Some wanted written recommendations (to stay at home or around safety needs at work) from a midwife/healthcare provider to take to their employer.

❖ **PARTNER AND HOUSEHOLD**

a) **Practical and emotional impacts:** Who a woman lives with had a big impact on her ability to stay safe, and on her emotional wellbeing. Many reported having supportive and helpful partners who could take care of essential shopping or other activities so they could stay at home. Women whose partners were away (and staying away due to social distancing) found it emotionally challenging. Pregnant women living alone (or only with small children) were particularly vulnerable to isolation and lacking practical support.

[redacted] PIP STUDY PREGNANT IN A PANDEMIC [redacted]

b) **Risk and others in the house:** Some women were confused that they had been placed in a risk category but members of their household had not, saying about partners "If he gets it, I get it" and worrying about household members going to shops or out to work. Co-parenting women felt unclear about guidelines and safety about children going between parents' households, and women with small children found it difficult to maintain social distancing when out with them. Women worried about lockdown easing and children returning to school. Some were shielding to protect a 'high risk' family member at home.

❖ FAMILY AND FRIENDS

Pregnant women were strongly affected by the loss of contact with family and friends, reporting "being pregnant is the time when you want family around you". Most reported friends and family as helpfully supportive of social distancing, but where they were not, women felt forced to cut some contacts. Either way, women experienced acutely the loss of normal contact with their social support systems at a critical period, including missing connection and the joy of pregnancy.

❖ THE CHALLENGE OF ESSENTIAL SHOPPING

Shopping was a key challenge for pregnant women, for regular food shopping and essentials (e.g. nappies for children) and to prepare for the coming baby. Being unable to maintain social distancing due to others' behaviour in shops caused anxiety. Some but not all women were able to access online shopping, vulnerable people timeslots or have partners do the shopping (though several worried about the safety of this). Being unable to get on the government's 'high risk' list (despite having comorbidities/higher risk factors) was a key concern/barrier for some. Women who were shielding relied on friends or charities for shopping. Many were taking extra precautions, such as disinfecting shopping before storing it.

❖ SAFETY OF GOING OUTSIDE

Concerns about Covid-19 meant women were limiting time outdoors with some not going out at all. Women's immediate environment made a big difference, with those in busy areas without easy access to open/green space feeling less safe going outside, as it was difficult to keep a distance from others. Women without gardens and those who were not going out seemed particularly at risk for negative mental health consequences as well as finding adhering to social distancing more challenging.

Information needs:

- While recognising that services were having to do their best in a difficult time, women expressed a need for more time, support and reassurance from midwives. Women had multiple anxieties, and some were very isolated, meaning their relationship with their midwife was especially important.
- Women also wanted written information from credible sources, with clear messages presented in an accessible format. While some wanted lots of detail, others were experiencing information overload

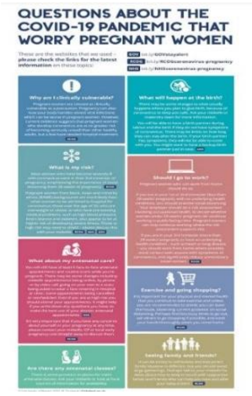

Information Infographic to share with pregnant women

To help meet women's information needs, we distilled women's main concerns from our findings into an infographic to share with pregnant women (in online formats (GIF and PDF) and/or as a printable leaflet). The aim is to help answer key questions, direct women to credible, up-to-date information resources and to facilitate conversations with midwives. The following (regularly updated) websites informed our infographic, and were included as interactive links for women to access:

**GOV**	bit.ly/**GOVstayalert**
**RCOG**	bit.ly/**RCOGcoronavirus-pregnancy**
**NHS**	bit.ly/**NHScoronavirus-pregnancy**

This report was produced from the initial findings from the 'Pregnant in a Pandemic' (PiP) Study. For more information, see: ***bristol.ac.uk/pip-study.*** *This work was supported by the* [redacted]*, the Wellcome Trust ISSF3 grant 204813/Z/16/Z and the Economic and Social Research Council ES/T501840/1.* [redacted]
